# Equine herpesvirus 1 myeloencephalopathy incidence and case fatality rates in the USA

**DOI:** 10.1093/jvimsj/aalag271

**Published:** 2026-10-29

**Authors:** Albert Picado, Alice Mundt, Gessica Tore, Aissatou Diawara, Tobias Warnken, Jeroen Kortekaas

**Affiliations:** Global Innovation, Boehringer Ingelheim Animal Health France, Saint-Priest, France; Global Innovation, Boehringer Ingelheim Vetmedica GmbH, Ingelheim, Germany; Global Innovation, Boehringer Ingelheim Animal Health France, Saint-Priest, France; Global Innovation, Boehringer Ingelheim Animal Health France, Saint-Vulbas, France; Global Strategic Marketing, Boehringer Ingelheim Vetmedica GmbH, Ingelheim, Germany; Global Innovation, Boehringer Ingelheim Animal Health France, Saint-Priest, France

**Keywords:** equine herpesvirus 1, equine herpesvirus myeloencephalopathy, EHM, EHV-1, surveillance data

## Abstract

**Background:**

Equine herpesvirus 1 (EHV-1) myeloencephalopathy (EHM) has substantial animal welfare and economic consequences. Despite extensive research, population-level estimates of EHM incidence and case fatality rates remain poorly characterized.

**Hypothesis/Objectives:**

Describe the burden of EHM and estimate EHM attack and case fatality rates in the United States.

**Animals:**

None.

**Methods:**

A retrospective observational study was conducted using surveillance data collected by the Equine Disease Communication Center (EDCC) in the United States during 2019-2023. Regression models were used to estimate the mean annual EHM incidence and to quantify attack and fatality rates. The incidence and potential seasonality of EHM by age category were also assessed.

**Results:**

Surveillance data showed that isolated EHM cases predominated, and the estimated mean annual incidence in the United States was 6.7 per million horses (95% CI, 4.9-8.9). The attack rate was 4.3% (95% CI, 3.5-5.4) and the case fatality rate was 49.5% (95% CI, 40.5-60.5). In the United States, older horses (>19 years) had higher EHM incidence than younger horses. Cases were more frequent from January to June, peaking in March.

**Conclusions and clinical importance:**

Equine herpesvirus 1 myeloencephalopathy is an uncommon clinical manifestation of EHV-1 infection, but the high case fatality rate emphasizes the clinical relevance of this condition.

## Introduction

Equine herpesvirus 1 (EHV-1) myeloencephalopathy (EHM) in horses is often described as an uncommon^1^ or rare^2^ disease, but the manifestation, often reported as outbreaks, can have substantial impact on animal health and economies. As an example, an EHM outbreak during a horse jumping event in Valencia, Spain, resulted in 114 EHM cases in 10 countries. At least 20 horses were euthanized, and sport horse events were canceled in 12 countries.^2^^,^^3^ Despite the impact of EHM, key epidemiological measures, including population-level incidence and case fatality rates remain poorly characterized.

Equine herpesvirus 1, recently renamed *Varicellovirus equidalpha 1*, is an enveloped double-stranded DNA virus that establishes a life-long persistent infection (latency) that is dormant but can be reactivated leading to productive viral replication and disease.^4^^,^^5^ The virus is endemic across the globe and typically causes upper respiratory tract infection. In pregnant mares, it is associated with late-gestation abortions. A variable, but usually small proportion of EHV-1 infected horses develops EHM manifesting with neurologic signs including ataxia, weakness, recumbency, lethargy, and urinary incontinence.^6^ A substantial number of horses suffering from EHM will die or be euthanized, whereas others may recover but can suffer from neurologic sequelae.^7^ Current EHV-1 vaccines do not confer protection against EHM.^6^^,^^8^

Equine herpesvirus 1 myeloencephalopathy has been reported for decades, but recent reports indicate that EHM incidence may be increasing.^6^ Extensive research has been conducted on EHM pathogenesis,^9^ clinical characteristics,^10^ sequelea,^7^ and treatments.^11^ The disease has been linked to neuropathogenic EHV-1 strains,^6^ but the risk factors^12^ associated with EHM development have not been fully characterized. Despite the growing body of literature on EHM, robust population-level estimates of key epidemiological indicators, such as incidence, attack rate, and case fatality rate, remain scarce. Estimating these variables will help determine the burden of EHM. We used surveillance data from the United States to estimate these key EHM epidemiological indicators.

## Materials and methods

### Data

A retrospective observational study was conducted using surveillance data reported to the Equine Disease Communication Center (EDCC) in the United States from 2019 to 2023. The EDCC is a web-based surveillance platform established to disseminate infectious disease information on horses in North America. Reports are submitted by veterinarians and are based on cases diagnostically confirmed by a State Animal Health Official or an attending veterinarian. The study included EDCC reports documenting EHM cases.

Data were gathered on EHM events (defined as one or more EHM cases on a premise or facility), EHM cases, horses at risk, and deaths attributed to EHM, as defined in Table 1. Equine herpesvirus 1 myeloencephalopathy is a reportable animal disease in the United States according to the Animal and Plant Health Inspection Service national list.^14^ However, notification requirements and compliance vary across states.^15^ The primary outcomes were the EHM attack rate, EHM case fatality rate, spatial and temporal distribution of EHM cases, age-adjusted EHM incidence, and annual EHM incidence per million horses.

**Table 1:** Definitions of the variables gathered from the EDCC database.

**Variable**	**Definition**
**EHM case**	A horse displaying neurologic signs (eg, ataxia, limb weakness, lethargy, and incontinence) with evidence of EHV-1 infection or a horse displaying neurologic signs exposed to EHV-1-infected horses.^13^
**EHM event**	Detection of one or more EHM cases on a premises or facility (ie, farm, veterinary hospital, and boarding facility) where there is confirmation of EHV spread or evidence of potential for virus transmission from an EHM case to susceptible horses on the premises.^13^
**EHM attributed deaths**	EHM cases succumbing to the infection or euthanized.
**Animals at risk**	Total number of horses present on the premises when the EHM event occurred.

Abbreviations: EDCC = equine disease communication center; EHM = equine herpesvirus 1 myeloencephalopathy; EHV-1 = equine herpesvirus 1.

### Analyses

The mean EHM attack rate was estimated using a negative-binomial generalized linear model (GLM), with the log number of horses at risk included as an offset. The mean EHM case fatality rate was estimated using a Poisson GLM, with the log number of EHM cases included as an offset. Corresponding 95% CIs were estimated using profile likelihood and Wald methods, respectively.^16^^,^^17^

The spatial distribution of EHM cases was represented in relation to the estimated horse population by state. Annual horse populations were extrapolated from American Horse Council figures for 2016 and 2023 by applying a global trend of −1.22% per year.^18–20^ Temporal distribution and seasonality were assessed using an epidemic curve and a negative-binomial GLM including month and year. As a secondary analysis, temporal differences were assessed by half-year.

Age-adjusted incidence was estimated using the available age of EHM cases and the USA horse population distribution by age group in 2015.^21^ Annual incidence was calculated using year-specific horse population estimates and exact Poisson CIs. Mean annual incidence was estimated using a Poisson generalized linear mixed model with a random intercept for year and horse population as an offset. As a secondary analysis, this model was restricted to states reporting at least one EHM case. All analyses were conducted using R version 4.2.2. Detailed methods are provided in the Appendix.

## Results

In total, 170 EHM events and 233 EHM cases were included in the analyses. Most EHM events (81%) only reported a single EHM case and 93% of them reported 1 or 2 EHM cases (Table 2). The mean number of EHM cases per event was similar across the study period: 1.2 in 2019, 1.4 in 2020, 1.3 in 2021, 1.6 in 2022, and 1.2 in 2023.

**Table 2:** Number of EHM cases and horses at risk per EHM event the United States (2019-2023).

**EHM cases/event**	**1 (%)**	**2 (%)**	**3-7 (%)**	**More than 7 (%)**	**Total**
**Frequency**	137 (81)	21 (12)	10 (6)	2 (1)	170
**Horses at risk/event**	**1-10 (%)**	**11-30 (%)**	**31-100 (%)**	**More than 100 (%)**	**Total**
**Frequency**	19 (17)	40 (35)	46 (40)	10 (9)	115

Abbreviation: EHM = equine herpesvirus 1 myeloencephalopathy.

The number of horses at risk was reported in 115 EHM events (68%). The estimated mean EHM attack rate was 4.3% (95% CI, 3.5%-5.4%). The number of EHM attributed deaths was reported in 148 EHM events (87%), including 69 EHM events (47% of the EHM events with mortality data) where zero deaths were recorded. The mean EHM case fatality rate was estimated to be 49.5% (95% CI, 40.5%-60.5%).

Figure 1 shows the spatiotemporal distribution of EHM cases in the United States from 2019 to 2023. The EHM cases were reported across 34 states (excluding Alaska and Hawaii). California and Virginia reported the largest number of EHM cases during the study period; 64 and 22 cases, respectively. Fifteen states did not report any EHM cases from 2019 to 2023 (Figure 1, top panel). The monthly epidemic curve (Figure 1, bottom panel) shows cases throughout the study period, with a surge in March 2022.

**Figure 1:**
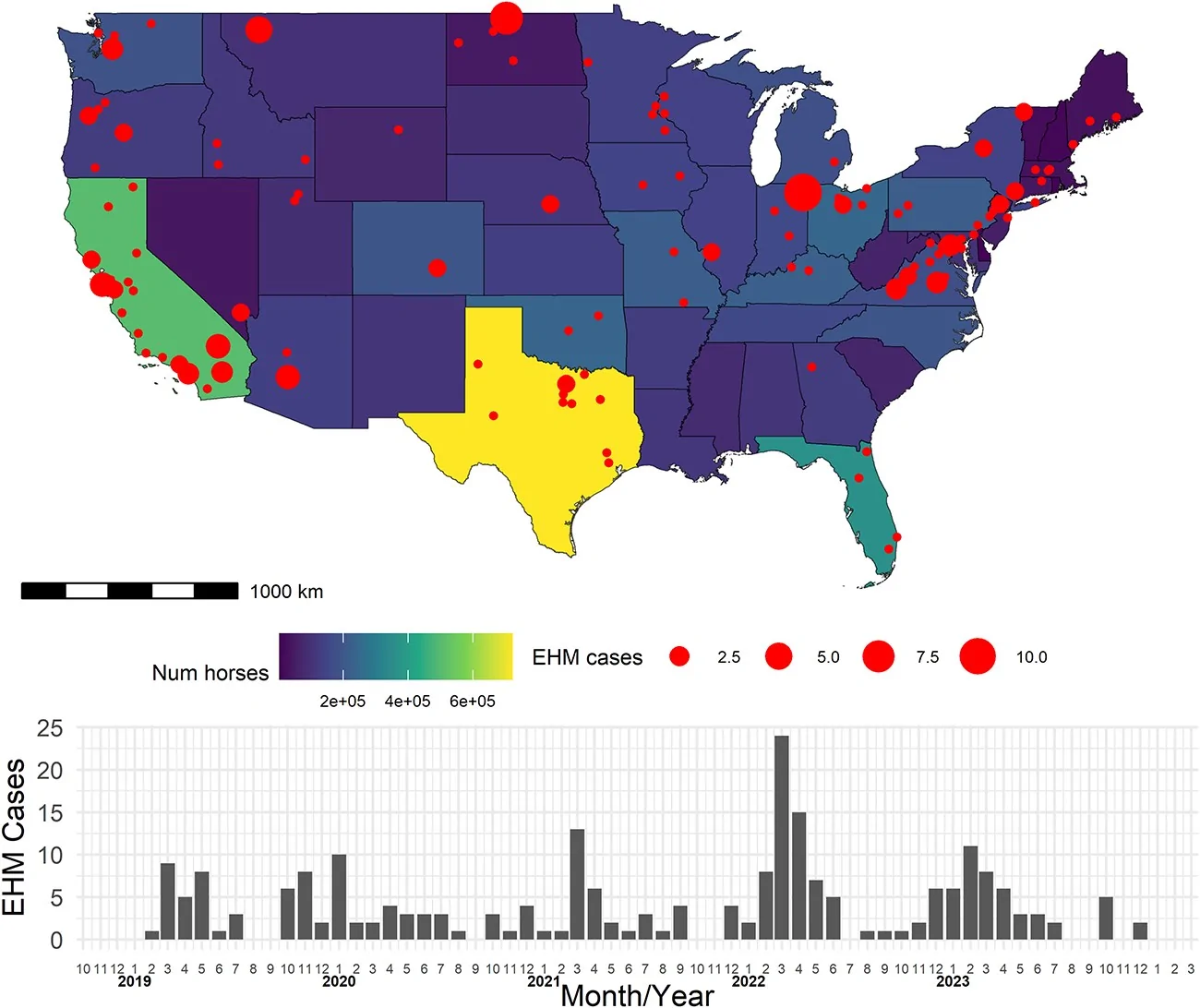
Spatial and temporal distribution of reported EHM cases in the United States, 2019-2023. Top: Map showing the locations of reported EHM cases over the study period. Bottom: Monthly epidemic curve (bars) of EHM cases from January 2019 to December 2023. Abbreviation: EHM = equine herpesvirus 1 myeloencephalopathy.

The EHM cases were reported throughout the year, with evidence of seasonality (Figure 2). Relative to January, March had a higher expected number of cases (incidence rate ratio [IRR] = 2.75), whereas August (IRR = 0.15) and September (IRR = 0.26) were significantly lower. In the half-year comparison, the expected number of EHM cases from January to June was 5.42 cases (95% CI, 4.00-7.34), significantly higher than the expected number of cases from July to December (2.10 EHM cases; 95% CI, 1.47-3.01).

**Figure 2:**
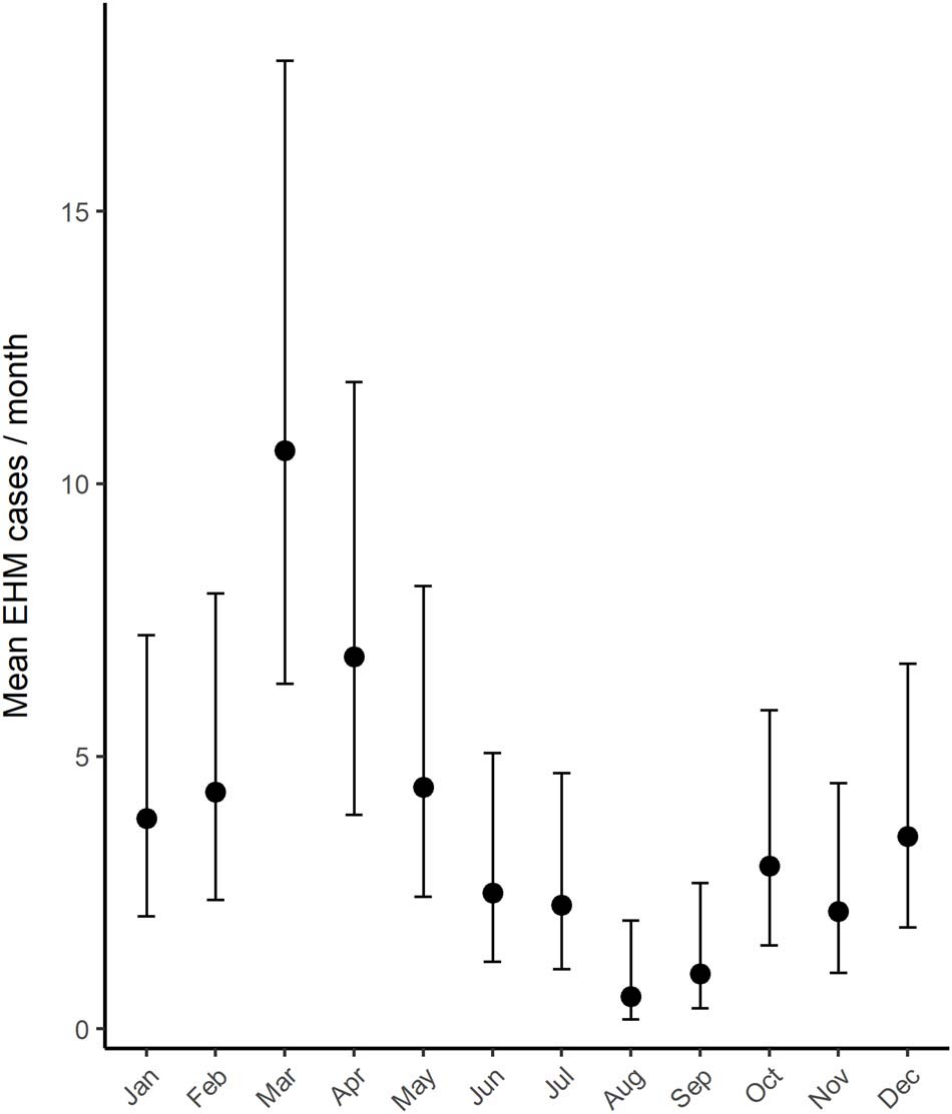
Expected monthly reported EHM cases in the United States (2019-2023) with 95% CIs, estimated from a negative-binomial model with month and year as fixed effects. A peak is evident in march and a decrease in August–September. Abbreviation: EHM = equine herpesvirus 1 myeloencephalopathy.

The estimated horse populations in the United States ranged from 6 984 296 in 2019 to 6 649 999 in 2023 and an average of 6 816 123 during the study period. The age of horses suffering from EHM was reported in 130 cases (56% of the EHM cases). The median age of EHM cases was 17 years old (IQR, 10-20). Most EHM cases with known age occurred in horses 5-19 years (56%) and > 19 years (37%); 7% were 0-4 years. The age-adjusted annual EHM incidence per million horses increased with age: 0-4 years, 1.1 (95% CI, 0.5-2.2); 5-19 years, 3.2 (95% CI, 2.5-4.1); > 19 years, 12.8 (95% CI, 9.4-17.0; Figure 3).

**Figure 3:**
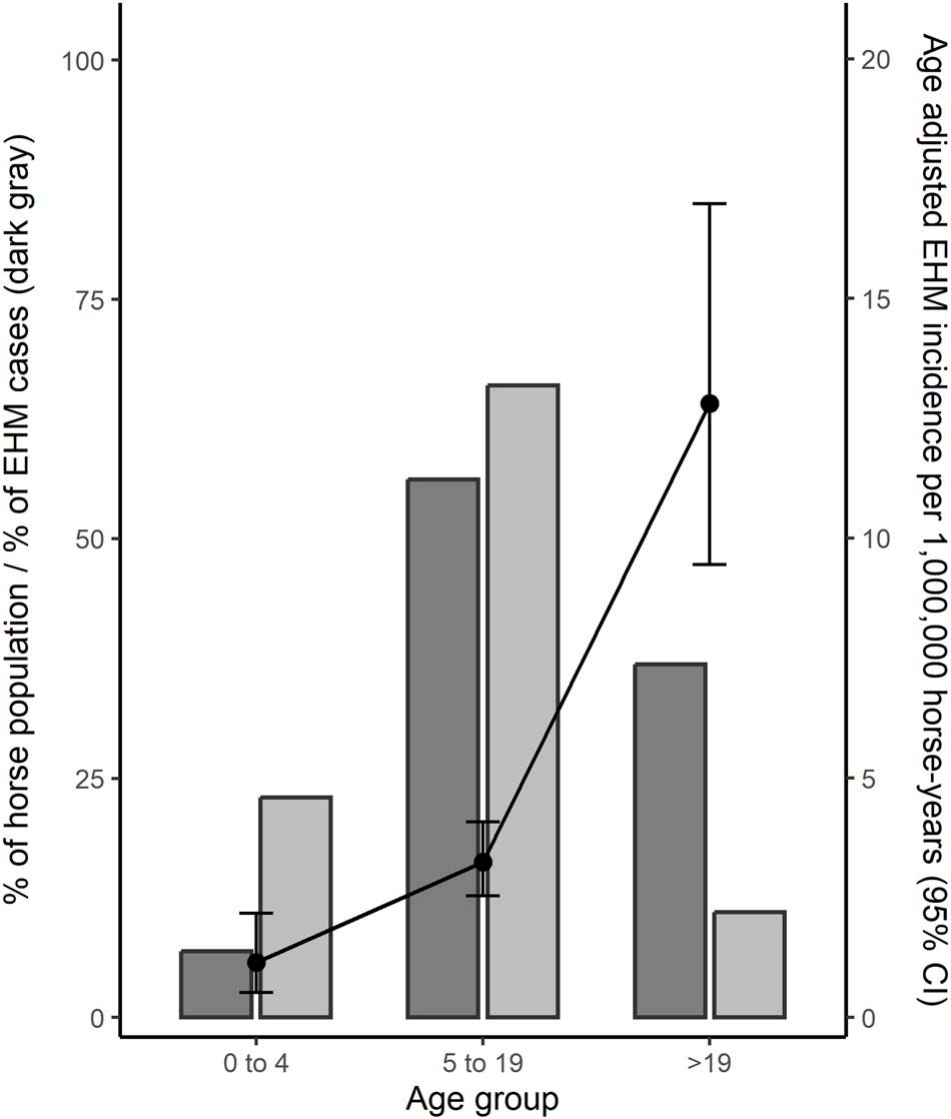
Left y-axis: Age distribution of horses and EHM cases in the United States. Bars show the percentage of the horse population (light gray) and of EHM cases (dark gray) by age group: 0-4, 5-19, and > 19 years. Right y-axis: Black points with whiskers show the age-adjusted annual EHM incidence per million horses and its 95% CI. Abbreviation: EHM = equine herpesvirus 1 myeloencephalopathy.

In the United States, annual EHM incidence was stable from 2019 to 2021 (5-6 cases per million horses) and increased significantly in 2022 to 10.7 per million horses (Figure 4).

**Figure 4:**
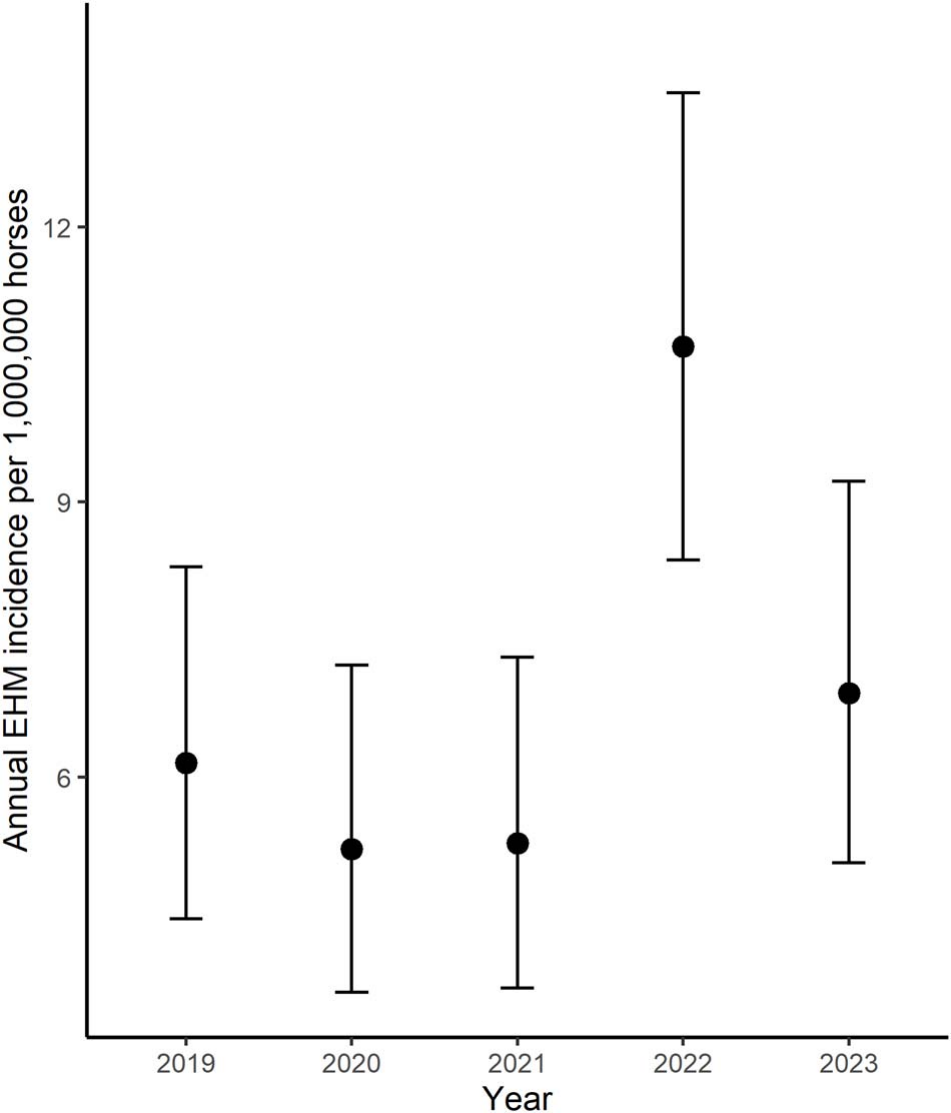
Annual EHM incidence in the United States, 2019-2023 (per million horses). Points show yearly estimates; error bars denote 95% CIs. Abbreviation: EHM = equine herpesvirus 1 myeloencephalopathy.

The mean annual EHM incidence rate in the United States was 6.7 cases per million horses (95% CI, 4.9-8.9). Limiting the dataset to the 34 states that reported at least one EHM case during the study period (average annual horse population 5.54 million) yielded a mean annual incidence of 8.2 EHM case per million horses (95% CI, 6.4-10.5).

## Discussion

Our study supports the hypothesis that EHM is a rare disease, with a country-wide annual incidence of 6.7 EHM cases per million horses based on the surveillance data from the United States. However, this figure may underestimate the actual burden of EHM. First, the number of EHM cases is likely to be underreported because of the reporting system. In the United States, EHM is specifically listed as a reportable disease in some states such as California^22^ but in others, such as Arkansas, only EHV-1 infections but not EHM are identified as reportable^23^ and not all EHM cases may be reported to EDCC. The underreporting of cases also may be exacerbated by the fact that, according to our results, the majority (>80%) of EHM cases are isolated cases, which may limit diagnosis and reporting. Large EHM outbreaks are more likely to be reported than small-scale outbreaks and isolated cases.^24^^,^^25^ Second, the number of EHM cases reported varies substantially across states. Thus, using country-wide incidence estimates likely underestimates the impact of EHM. When restricting the analysis to the 34 states that reported EHM cases, the mean annual incidence rate increased to 8.2 per million horses, yet remained low. The EHM burden likely remains underestimated in other countries as well, because EHM notification often relies on passive epidemiological surveillance programs.^26^

To correctly assess the impact of EHM, other epidemiological indicators also should be considered. The 49.5% case fatality rate estimated from the surveillance data is higher than previously reported estimates: 12% (11/89) in the 2021 EHM outbreak in Valencia, Spain^27^ and 18% (2/11) in a recent outbreak in Chile,^28^ for example. Although comparable case fatality rates have been described in a few outbreaks: 42.9% (6/14) in a group of aged horses in the United States^29^ and 46.7% (7/15) in an EHM outbreak on a stud farm in New Zealand,^30^ our estimate is based on a larger dataset (148 EHM events in 5 years). Comparisons between surveillance-derived estimates and those reported in the literature should be interpreted with caution, because they originate from different data sources, study designs, and epidemiological contexts and are therefore not directly comparable. Nevertheless, the estimate from the surveillance data likely underestimates the actual case fatality rate because the follow-up of EHM cases is often limited. Thus, horses that died or were euthanized because of EHM sequelae after the event was reported to the EDCC may not have been registered in the platform and thus they may not have been included in the calculations. Reported deaths frequently reflect euthanasia decisions, which often are made for horses with severe neurologic signs. Factors such as age, clinical severity, and disease control measures are likely to influence the risk of death or euthanasia in EHM cases, but these variables could not be evaluated in our study. Even if EHM incidence is low, the high case fatality rate of EHM warrants the development of specific preventive tools for this disease.

Our study identified other interesting epidemiological features. Isolated cases of EHM predominate, and they are associated with a low attack rate (4.3%). This contrasts with attack rates reported in EHM outbreaks. The proportion of EHM cases ranged from 10.6% (7/66) in France^31^ to 19.2% (20/104) in Canada^32^ and 34.1% (46/135) in the United States.^33^ This finding suggests that individual host factors are the main drivers for the development of EHM,^25^^,^^34^ including the immunological response to EHV-1 infection.^34^ The fact that EHM cases are sporadic supports the hypothesis that EHM is associated with reactivation of latent EHV-1 infection in the same horse.^4^^,^^5^^,^^25^

The incidence of EHM per year shows a peak of cases in 2022. This increase was associated with a higher number of EHM events (45 in 2022 compared with 25 in 2020), but a similar mean number of EHM cases per event (1.6 in 2022 vs 1.4 in 2020). The surveillance data do not allow us to determine whether the increase in incidence in 2022 reflects a true increase in cases or reporting bias linked to recent EHM outbreaks^2^^,^^35^ or to other events. For example, a previous study hypothesized that the significant increase of equine influenza case the in United Kingdom in the period 2021-2024 compared with 2020 may have been associated with the resumption and intensification of horse movements and gatherings after the disruption of equestrian activities in 2020 because of COVID-19 restrictions.^36^ Lifting COVID-19 restrictions similarly may have increased EHV-1 transmission by facilitating the resumption of horse movements and gatherings. However, this hypothesis and other potential explanations for the observed increase were not evaluated in our study.

Cases of EHM are reported throughout the year, but their incidence seems to be seasonal with higher numbers of cases in the first part of the year and a peak in March. The seasonality of EHM has been reported previously; in the Netherlands the risk of EHM was associated with late fall, winter, and spring seasons.^25^ Evaluating temporal trends of EHV-1 infections may help evaluate the relationship between EHV-1 infection and EHM occurrence. The data collected by surveillance platforms such as EDCC could be used to conduct these analyses.^26^

As shown by the surveillance data, EHM is reported in horses from less than 1 to 33 years of age. However, EHM often is identified as a disease that tends to affect older horses (>20 years),^37^ with the risk for neurologic disease increasing with age.^12^^,^^25^^,^^29^ This observation was confirmed by the surveillance data in the United States where EHM incidence increased with age and peaked in horses that were over 19 years old.

The data from the EDCC have some limitations. Other than the potential underreporting and reporting bias pointed out earlier, the lack of or limited information on some key features impeded conducting some analyses. For example, the lack of data on the sex and the breed of the EHM cases precluded evaluating the hypothesis that EHM incidence may be associated with these 2 factors.^12^^,^^25^^,^^34^^,^^38^ Nevertheless, our study demonstrates that surveillance data allow estimating the impact of diseases in horses. Similar surveillance systems are available in other countries and have been used to study the epidemiology of infectious pathogens in horses in the Netherlands,^26^ the United Kingdom,^36^ and France.^39^ Standardizing and facilitating access to such data would help maximize the use of these databases for epidemiological studies.

Surveillance and reporting of EHM should be improved, and further research is needed to understand the factors associated with EHM in individual cases and outbreaks and to develop effective preventive and control tools.

## Data Availability

The data analyzed in this study are publicly available through the Equine Disease Communication Center (EDCC) outbreak alerts website: https://equinediseasecc.org/alerts.

## Off-label antimicrobial declaration

The authors declare no off-label use of antimicrobials.

## Institutional animal care and use committee or other approval declaration

The authors declare no institutional animal care and use committee or other approval was needed.

## Appendix: Materials and methods

### Data

A retrospective observational study was conducted using surveillance data reported to the Equine Disease Communication Center (EDCC) in the United States from 2019 to 2023. The EDCC is a web-based surveillance platform established to disseminate infectious disease information on horses in North America. Reports to the EDCC are submitted by veterinarians and are based on cases that are diagnostically confirmed by a State Animal Health Official or an attending veterinarian. Reports typically include the disease, date, county, state, case status, and, when available, the numbers of confirmed, exposed and dead horses.

From the EDCC, data on EHM events (defined as one or more EHM cases on a premise or facility), EHM cases, horses at risk, and deaths attributed to EHM as defined in Table 1 were gathered. It is a reportable animal disease in the United States according to the Animal and Plant Health Inspection Service (APHIS) national liste.^14^ However, the notification requirements and compliance of EHM cases vary across states.^15^

### Analyses

#### EHM attack- and case fatality rates

The surveillance data were analyzed to illustrate the characteristics and temporal distribution of EHM cases in the United States from 2019 to 2023. The number of EHM cases and horses at risk per event were used to estimate the mean EHM attack rate defined as the mean incidence of EHM cases in an EHM event (EHM cases/animals at risk). A negative-binomial generalized linear model (GLM) with the log number of horses at risk included as an offset was used to estimate the mean EHM attack rate and its 95% CI. The 95% CI was estimated using the profile likelihood method.^16^ A similar analytical approach was used to estimate the mean EHM case fatality rate defined as the mean proportion of EHM attributed deaths among EHM cases in an EHM event (EHM attributed deaths/EHM cases). A Poisson GLM with the log number of EHM cases included as an offset was used to estimate the mean EHM fatality rate. The 95% CI was estimated using the Wald method.^17^

#### EHM cases distribution

To represent the spatial distribution, the EHM cases in the United States were plotted on a country map representing the mean horse population per state (excluding Hawaii and Alaska) during the study period. The horse population per state in the United States is estimated periodically by the American Horse Council (AHC). To estimate the annual horse population per state in the United States from 2019 to 2023, the AHC figures for 2016 (7.246 million) and 2023 (6.65 million) were used to extrapolate the population during the study period, applying a global trend of −1.22% per year.^18–20^ An epidemic curve was used to represent the temporal distribution of EHM cases over the study period.

The total number of EHM cases was aggregated by calendar month. Seasonality was assessed using a negative-binomial GLM with month and year included as explanatory variables. The expected cases per month and their 95% CIs were obtained as estimated marginal means (EMMs). Monthly differences were expressed as incidence rate ratios (IRRs) using January as the reference. Incidence rate ratios values > 1 indicate more cases than January and IRR values < 1 indicate fewer. As a secondary analysis, an analogous model was fit with half-year as the explanatory variable (H1: January–June; H2: July–December).

#### EHM incidence

The age-adjusted EHM incidence was estimated using the data available on the age of the EHM cases from the surveillance databases and the distribution of the horse population by age groups in 2015 in the United States^21^ as a reference. It was assumed that the age distribution was similar during the study period. Based on the available data the age groups considered were: 0-4 years (23%), 5-19 years (66%), and 20 years or older (11%). For each age group, the annual EHM incidence per million horses was estimated and 95% CIs were calculated using exact Poisson intervals.

To evaluate changes in EHM incidence across the study period, annual EHM incidence per million horses was computed as the number of reported cases divided by the corresponding year-specific horse population. Corresponding 95% CIs were calculated using the exact Poisson and plotted alongside the yearly estimates.

The mean annual incidence rate per million horses was estimated using a Poisson generalized linear mixed model (GLMM) with log link, a random intercept for year, and a log-exposure offset for the horse population expressed in millions of horses. As a secondary analysis, the model was repeated after restricting the dataset to states that reported at least one EHM case during the study period. The 95% CI were computed on the log scale and back-transformed to the original scale.

#### Data availability and statistical software

The database used in the analyses is available on request. All the analyses were conducted using R version 4.2.2. The different packages used are listed as follows: glmmTMB, sf, ggplot2, lubridate, tidyr, dplyr, gridExtra, lme4, and RColorBrewer.

1. Brooks, M. E., Kristensen, K., van Benthem, K. J., Magnusson, A., Berg, C. W., Nielsen, A., … & Bolker, B. M. (2017). glmmTMB balances speed and flexibility among packages for zero-inflated generalized linear mixed modeling. *The R Journal*, 9(2), 378-400.
2. Pebesma, E. (2018). Simple Features for R: Standardized Support for Spatial Vector Data. *The R Journal*, 10(1), 439-446.
3. Wickham, H. (2016). ggplot2: Elegant Graphics for Data Analysis. *Springer-Verlag New York*.
4. Grolemund, G., & Wickham, H. (2011). Dates and Times Made Easy with lubridate. *Journal of Statistical Software*, 40(3), 1-25.
5. Wickham, H., & Henry, L. (2023). tidyr: Tidy Messy Data. R package version 1.3.0.
6. Wickham, H., François, R., Henry, L., & Müller, K. (2023). dplyr: A Grammar of Data Manipulation. R package version 1.1.2.
7. Auguie, B. (2017). gridExtra: Miscellaneous Functions for “Grid” Graphics. R package version 2.3.
8. Bates, D., Mächler, M., Bolker, B., & Walker, S. (2015). Fitting Linear Mixed-Effects Models Using lme4. *Journal of Statistical Software*, 67(1), 1-48.
9. Neuwirth, E. (2014). RColorBrewer: ColorBrewer Palettes. R package version 1.1-2.

## Abbreviations

AHC: American Horse Council
EDCC: Equine Disease Communication Center
EHM: equine herpesvirus 1 myeloencephalopathy
EHV-1: equine herpesvirus 1
IRR: incidence rate ratio
